# Assessment of Common Food Allergens in Infants With Atopic Dermatitis in Kashan From 2005 to 2021

**DOI:** 10.1002/iid3.70544

**Published:** 2026-10-06

**Authors:** Seyyed Ramin Madani, Mahdi Salehi, Marzieh Heidarzadeh Arani, Zahra Shaterian, Mohammad Mahdi Heidari, Mohammad Javad Azadchehr

**Affiliations:** ^1^ Department of Pediatrics, Faculty of Medicine Kashan University of Medical Sciences Kashan Iran; ^2^ Student Research Committee, Faculty of Medicine Kashan University of Medical Sciences Kashan Iran; ^3^ Pediatric Nephrology Department, Aliasghar Hospital Iran University of Medical Sciences Tehran Iran; ^4^ Department of Pediatrics, Faculty of Medicine Iran University of Medical Sciences Tehran Iran; ^5^ Clinical Research Development Unit, Shahid Beheshti Hospital Kashan University of Medical Sciences Kashan Iran; ^6^ Infectious Diseases Research Center Kashan University of Medical Sciences Kashan Iran

**Keywords:** atopic dermatitis, cow's milk, egg, food allergy, infant, skin prick test

## Abstract

**Introduction:**

Infants with atopic dermatitis (AD) are more likely to experience food allergies, and AD children experience asymptomatic food sensitivity. Therefore, this study aimed to evaluate food allergies in infants with AD with different tests during 17 years.

**Method:**

This retrospective case‐series study included 98 infants with AD who underwent clinic prick skin tests from 2005 to 2021. After recording demographic features, all food allergens from forearm skin scratch tests for specific allergens were recorded from medical files. The data were then collected, entered into SPSS v24, and analyzed.

**Results:**

The majority of infants (66.3%) had mild dermatitis, and 9 cases (9.18%) had severe AD. Infants with AD were most allergic to egg (64.3%), cow's milk (49%), nuts (35.7%), soy (20.4%), and wheat (12.2%). We found that egg allergy was significantly higher in girls than boys (74.1% vs. 52.3%, *p* = 0.025). Moreover, we found that allergy to cow's milk was significantly higher in infants with an age at onset of clinical manifestations lower than 6 months than those with an age at start greater than 6 months (56.06% vs. 34.3%, *p* = 0.044). Moreover, age at onset of clinical manifestations in severe AD cases was significantly lowest compared to other severity levels (2.78 ± 2.77 months) (*p* = 0.014).

**Conclusion:**

Infants with AD were most allergic to egg and cow's milk. Medicine, diet, and avoiding food allergies may help infants with AD, especially avoiding eggs and cow's milk in dietary before 6 months.

## 1 Introduction

Atopic dermatitis (AD), or eczema, is the most common recurrent skin disease during infancy and childhood [1]. AD affects 10%–30% of children worldwide and often occurs in families with other atopic diseases, such as asthma, allergic rhinitis, and food allergies [2]. AD is a chronic skin inflammatory allergic condition that causes itching, dryness, redness, lichen, peeling, macules, vesicles, and papules to be the prominent symptoms of this illness, which vary by person [3]. Infants with AD are more likely to develop allergic rhinitis or asthma later in childhood [4]. AD causes sleep difficulties, irritability, psychological and social status issues, low quality of life, and significant family expenses [5]. In addition to its many clinical signs, AD is more frequent in children (25%) than adults (2%–3%) [6]. Inappropriate and excessive immune system responses to harmless allergens are known as hypersensitivity or allergic reactions in AD [7]. Immunologically, AD is a chronic inflammatory disease with complex interactions between skin barrier dysfunction and immune dysregulation. It is mainly characterized by an impaired T helper 2 (Th2) immune response [8], leading to excessive production of interleukins such as IL‐4, IL‐5, and IL‐13, increased serum IgE levels, and activation of eosinophil and mast cells. These immune mechanisms play an important role in the persistence of inflammation and the development of allergic reactions early in life, linking AD to the later development of other atopic diseases, and cause serious side effects like cardiac side effects [9, 10].

Multiple reasons cause AD, such as genetics, environmental variables, such contact stimuli, hormones, stress, inhalation, and dietary allergies have a role [11, 12]. Food allergens are linked to severe AD in 30%–40% of individuals [13]. In certain individuals, food allergies accelerate illness development and severity [14]. Because immune sensitization early in life may influence long‐term allergic inflammation throughout life [15], identification of relevant food allergens in infancy is of clinical importance. However, because the spectrum of food allergen sensitivity varies based on various factors, including age, geographic region, and dietary patterns, long‐term data from specific populations remain limited. Review and study of region‐specific evidence using validated immunological methods is essential to guide targeted dietary interventions and avoid unnecessary immune stimulation resulting from experimental food exclusion [16]. Therefore, identifying food allergens in allergic illnesses is crucial due to the changing pattern of these allergens and the variation of cuisines in groups and countries [17].

Food allergen frequency in AD varies by age and area in Iran [18]. In IgE‐dependent allergic disorders, the skin prick test is a reliable way to detect these allergens. This affordable, consistent, minimally intrusive approach yields fast results [19]. Food allergens affect the occurrence and severity of dermatitis illness due to their differences in geography, culture, and age, as well as the need of understanding them to avoid it [2]. However, eliminating the effective dietary allergen may control the condition. Unfortunately, many physicians offer patients a list of dietary items to exclude from both the mother's and child's nutrition, causing double starvation and injury [17]. However, precisely identifying and eliminating food allergens controls disease symptoms and improves AD patients’ quality of life, therefore we chose to explore common food allergens from 2005 until 2021 in infants with AD by skin prick test, a minimally intrusive, affordable, and accurate approach that did not need food removal.

## 2 Methods and Materials

### 2.1 Target Group

This descriptive retrospective study was conducted in the asthma and allergy clinic of Kashan Shahid Beheshti Hospital in Iran from January 2005 to December 2021. The electronic files related to infants (children under 2 years old) with AD and skin prick tests to determine food allergens were reviewed and recorded. The inclusion criteria consisted of infants (age 6–24 months) with AD with completed medical files. Exclusion criteria consisted of infants with AD without the possibility of performing a skin prick test, drug use that cannot be stopped and disrupts the test results, severe skin disease other than AD, and a history of anaphylaxis to food. We also excluded patients with incomplete data.

### 2.2 Study Design

The study flowchart is shown in Figure 1. The files of 98 infants with AD based on inclusion and exclusion criteria were included. The study received ethics approval from the Ethics Committee of Kashan University of Medical Sciences (IR. KAUMS. NUHEPM. REC.1400.013). Informed consent was obtained from all parents.

**Figure 1:**
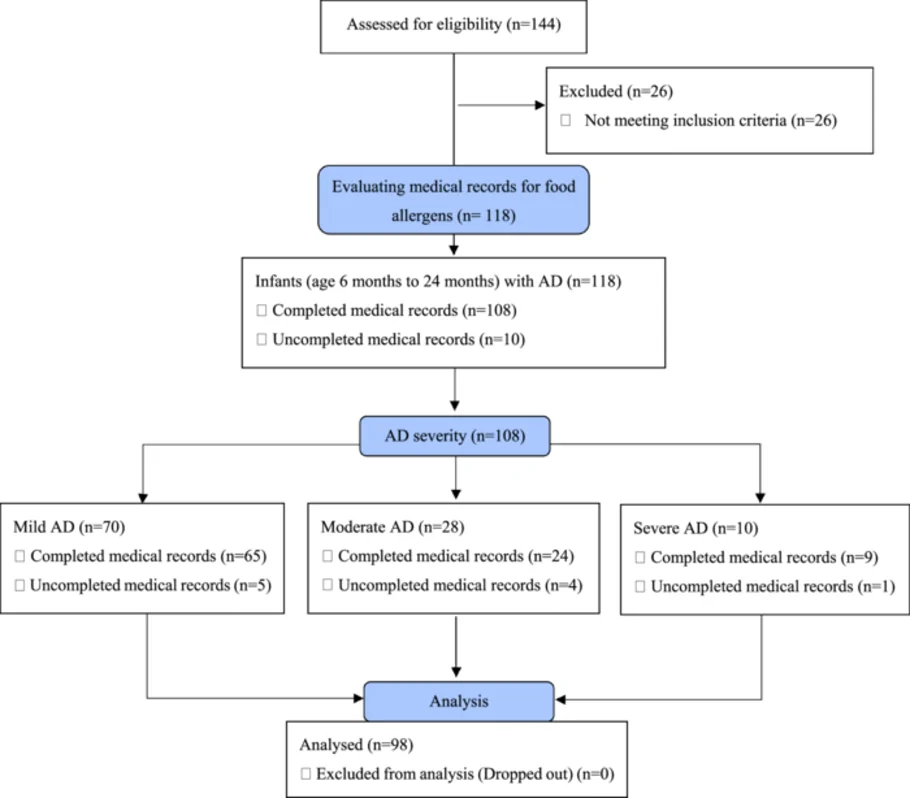
Study flowchart.

Demographic variables (gender, age) and type of food allergen (egg, milk, tree nuts, soy, wheat, seafood, rice, peanut), the age of onset of the disease, and the type of infant nutrition were recorded. It should be noted that the skin prick test is a valuable diagnostic test for different allergens. This test is considered positive if a swelling of more than 3 mm occurs at the site of the allergen skin scratch. To divide the allergy test in terms of severity, a skin reaction in the form of swelling of 3–5 mm was considered mild, 5–10 mm as moderate, and more than 10 mm as severe. The selected allergens for patients with AD were 8 important and common food allergens, including milk, eggs, tree nuts, soybeans, wheat, seafood, rice, and peanuts, and extracts from each were used as allergens and with a test. In the asthma and allergy clinic of Kashan Shahid Beheshti Hospital, extracts were injected into a skin scratch on the palm side of the patient's hand. The use of antihistamines (H1 and H2) and systemic steroids was stopped a week before injection. After 15–20 min of entering the extract into the skin, the entry site or the same scratch was checked. If they had erythema greater than 8 mm and induration greater than 3 mm, the test was considered positive.

### 2.3 Data Analysis

Data were analyzed and reported only for patients who completed the trial. Statistical analysis of data was performed using SPSS version 24 software (SPSS Inc., Chicago, IL, USA). Analytical analysis was done by Chi‐square and independent *t*‐tests, and ANOVA. The two tailed *p*‐value < 0.05 were considered significant.

## 3 Result

We included the cases of 98 infants with AD in this study. Girls had the highest frequency (55.1%). In the first 6 months of life, 63.3% of infants consumed only breast milk, 13.3% consumed only infant formula, and the rest consumed a combination. The average age of onset of AD among the studied infants was 5.72 ± 5.89 months (1–24 months). Also, the severity of dermatitis in most infants (66.3%) was mild, then moderate (24.5%), and severe (9.2%).

Egg (64.3%), cow's milk (49%), nuts (35.7%), soy (20.4%), and wheat (12.2%) were the most common food allergens among infants with AD, respectively.

The type of food allergens did not have a significant correlation with the type of nutrition in the first 6 months of life and severity of AD (*p* > 0.05), however, we found that allergy to egg was significantly higher in girls as compared to boys (74.1% vs. 52.3%, *p* = 0.025). Moreover, we found that allergy to cow's milk was significantly higher in infants with an age at onset of clinical manifestations lower than 6 months as compared to those with an age at start greater than 6 months (56.06% vs. 34.3%, *p* = 0.044). However, allergy to wheat in infants with age at onset of clinical manifestations lower than 6 months was five times more than those with age at start greater than 6 months (16.6% vs. 3.1%) but due to lower sample size in both groups, this difference was not statistically significant (*p* > 0.05) (Table 1).

**Table 1:** Distribution of food allergen types in AD subjects based on demographic variables.

Food allergens		Soy (*n* = 20)	Egg (*n* = 63)	Cow's milk (*n* = 48)	Wheat (*n* = 12)	Nuts (*n* = 35)
Variables						
**Sex (%)**	Girl (*n* = 54)	12 (22.2)	40 (74.1)	26 (48.1)	7 (13)	21 (38.9)
	Boy (*n* = 44)	8 (18.1)	23 (52.3)	22 (0.5)	5 (11.4)	14 (31.8)
***p*‐value**		0.621	**0.025**	0.855	0.81	0.467
**Type of nutrition in the first six months of life (%)**	Breast milk (*n* = 62)	13 (21)	40 (64.5)	30 (48.4)	7 (11.3)	(18.29)
	Formula (*n* = 12)	1 (7.7)	7 (53.8)	6 (46.1)	0 (0)	6 (46.1)
	Breast milk/Formula (*n* = 24)	6 (26.1)	16 (69.5)	12 (52.2)	5 (21.8)	11 (47.8)
***p*‐value**		0.496	0.884	0.988	0.436	0.187
**Severity of AD (%)**	Mild (*n* = 65)	16 (24.6)	43 (66.1)	36 (55.4)	9 (13.9)	20 (30.8)
	Moderate (*n* = 24)	4 (16.7)	13 (54.2)	7 (29.1)	2 (8.3)	11 (45.8)
	Severe (*n* = 9)	0 (0)	7 (77.8)	5 (5.5)	1 (11.1)	4 (44.4)
***p*‐value**		0.526	0.39	0.082	0.775	0.356
**Age at onset of clinical manifestations (months)**	< 6 months (*n* = 66)	15 (22.7)	43 (65.15)	37 (56.06)	11 (16.6)	24 (36.36)
	≥ 6 months (*n* = 32)	5 (15.6)	20 (62.5)	11 (34.3)	1 (3.1)	11 (34.3)
***p*‐value**		0.413	0.797	**0.044**	0.055	0.847

*Note:* Bold values indicate statistically significant differences, defined as *p* < 0.05. Specifically, the association between sex and egg allergy was statistically significant (*p* = 0.025).

By evaluating the severity of AD based on demographic features, we found that the age at onset of clinical manifestations in cases with severe AD was significantly lowest as compared to other severity levels (2.78 ± 2.77 months vs. 7.17 ± 5.46 and 5.60 ± 6.23 months for moderate and mild groups, respectively) (*p* = 0.014), however, we did not find significant differences in the terms of AD severity based on gender and type of nutrition in the first 6 months of life (*p* > 0.05) (Table 2).

**Table 2:** Distribution of AD severity in AD subjects based on demographic variables.

AD severity		Mild (*n* = 65)	Moderate (*n* = 24)	Severe (*n* = 9)	*p* value
Variables					
**Sex (%)**	Girl (*n* = 54)	34 (62.9)	16 (29.6)	4 (7.5)	0.384
	Boy (*n* = 44)	31 (70.4)	8 (18.2)	5 (11.4)	
**Type of nutrition in the first six months of life (%)**	Breast milk (*n* = 62)	40 (64.5)	16 (25.8)	9 (9.7)	0.414
	Formula (*n* = 12)	8 (61.5)	5 (38.5)	0 (0)	
	Breast milk/Formula (*n* = 24)	17 (74)	3 (13)	13 (13)	
**Age at onset of clinical manifestations (months)**		5.60 ± 6.23	7.17 ± 5.46	2.78 ± 2.77	0.014

## 4 Discussion

Our study showed that infants with AD were most frequently allergic to eggs, cow's milk, and nuts. Egg allergy was significantly more common among girls, whereas cow's milk allergy was significantly more common among infants whose age at onset of clinical manifestations was less than 6 months. Moreover, the age at onset of clinical manifestations was significantly lower in infants with severe AD than in those with less severe disease.

The prevalence of AD and food allergies has increased in recent decades, especially in developing countries. AD, with a prevalence of 33%–75%, is one of the most common skin diseases [20], which increases the sensitivity of the immune system to dealing with antigens that normally do not seem to be harmful. AD and food allergy are clearly related; the issue of food allergy as an aggravating factor of this disease has always been controversial [21]. Studies have shown that 30%–40% of children with moderate to severe AD have food allergies [22].

We found that girls have more risk for developing AD. In a study conducted on infants with AD, it was found that, among infants, the prevalence of AD was higher in girls. In a similar study conducted by Afshari et al. [23] on the prevalence of AD, they found that the incidence of AD among children is not related to the age and gender of the patients. Also, based on the similar results that Golchai et al. [24] and Ghaderi et al. [25] conducted on the demographic characteristics of patients with AD, they found that the age and gender of the patients have no relationship with having AD. The higher prevalence of autoimmune diseases such as AD in females justifies this problem in the general population.

Egg and cow's milk were the most common food allergens among infants with AD, with 64.3% and 49%, respectively. In a similar study, Chen et al. [26] performed a skin prick test on 1687 children between 0 and 2 years old, using different food extracts, and found allergies to eggs, shrimp, and fish in most cases. Ahanchian et al. [27], examining 207 children with AD, found that the highest frequency of sensitivity to food allergens was egg white (34.8%), egg yolk (26.2%), and cow's milk (16.9%). In the study conducted in Bushehr, Farrokhi et al. [28] found that peanuts, walnuts, and soybeans had the highest level of sensitivity, respectively. In another study by Salehi et al. [29], the most common allergens were eggs, milk, wheat, peanuts, and soy, respectively, which almost corresponds to the group of common allergens in this study. Čelakovská et al. [30] in the Czech Republic stated that among children with AD, the most common food allergens were nuts, milk, and peanuts. In the study of Farrokhi et al. [28], in children aged 6 months to 5 years with AD found that egg white, egg yolk, peanut, kiwi, strawberry, milk, walnut, fish, banana, and wheat were among the most common food allergens. The reason for the different findings of these studies can be due to the differences in dietary, cultural, and racial habits, as well as the age of the cases. Furthermore, higher consumption of a food item in a society increases the probability of developing allergies. In fact, due to the higher consumption of milk, eggs, and wheat among people in society, allergies to these substances are also more common in allergic patients. The most common food allergens in America, Europe, and the Middle East are cow's milk and eggs, and fruits, respectively. In a previous study in Iran, among allergic patients, the most common food allergies were milk, eggs, and wheat flour in a skin prick test [31]. In recent years, numerous factors have spread all forms of allergy illnesses worldwide [32]. When exposed to an allergen, B lymphocytes generate large levels of IgE. This occurs when TH2 cells activate and release Interleukin 4 (IL‐4), IL‐5, IL‐9, and IL‐13, which produce IgE. The allergic reaction is caused by IL‐4, which boosts IgE‐mediated immune responses [33]. IgE binds to mast cell and basophil ε Fc receptors after production. These cells activate and produce inflammatory mediators when linked to the allergen, causing allergy symptoms [34].

According to the findings of Dominiguez et al. [22] in 2020, which aimed to investigate the relationship between food allergies and AD, younger children, often compared to eggs, milk, or peanuts, show sensitivity, while adults are more sensitive to environmental allergens such as mites, house dust, molds, animal dander, or pollen. Dominguez et al. [22] also found that sensitivity to eggs decreases with age, which is expected due to the improvement of food sensitivities with age [35]. Because food sensitivities often improve with age [35]. In Pourpak et al. [36], cow's milk allergy had the highest prevalence in children under 6 years of age. For people under 1‐year‐old, milk and eggs have the highest prevalence. In 1–9 years, cow's milk, tomato, egg white, and veal meat were the most prevalent. Tomatoes, cow's milk, almonds, and veal meat have the highest prevalence among food allergens at the ages of 6–10 years. Allergies to milk and eggs have the highest prevalence. As can be observed, egg and milk allergies are the most common cases of food allergies in children of all ages, especially infant ages, which is due to the higher consumption and early initiation of these foods in children, especially at a younger age [36]. Therefore, the type of food allergen was related to the age of the patients. However, in this study, no correlation was found between the type of allergen and the severity of the allergy. These existing differences can be attributed to the difference in the age of the investigated patients and the difference in eating habits in different countries, and these factors will play an effective role in the distribution of the frequency pattern of allergens. Due to the differences in the geographical area of the study, the population under study, and even the year of the study, the weather, and the quality and quantity of food available to the mother and the child, the results obtained in the studies have clear and distinct differences from each other. That is why we decided to conduct this study in our region, and the smallness of our statistical population compared to other studies may have caused this difference.

We found that the type of nutrition in the first 6 months of life did not relate to food allergens or severity of AD; however, allergy to egg was significantly higher in girls, and cow's milk was significantly higher in infants with age at onset of clinical manifestations lower than 6 months. These findings are consistent with several studies that have failed to show a clear protective or predisposing effect of exclusive breastfeeding or formula feeding on food allergen sensitivity or AD severity [37, 38]. In a similar study conducted by Ghaderi et al. [25] with the aim of determining the risk factors of AD, they found that breastfeeding and receiving formula, or the age of starting feeding with complementary foods, had no effect on the incidence and severity of AD symptoms. Ghaffari et al. [39] stated that the age of onset of allergic reactions can be an influencing factor in the prevalence of food allergies in normal communities or among allergic diseases and AD. These findings support current evidence that infant feeding practices alone are not sufficient to explain allergic outcomes without considering genetic susceptibility and immune mechanisms. On the other hand, in our study, egg allergy was significantly more common in girls than in boys in our cohort. Although data on gender differences in food allergen sensitivity in infancy are limited, some studies have reported higher rates of IgE‐mediated egg sensitivity in female infants, potentially indicating gender differences in the regulation of the immune response [40, 41]. This observation warrants further investigation in larger cohorts.

Our study had some limitations. First, this study had a retrospective design and relied on information extracted from electronic medical records; designing prospective studies is necessary. Second, due to the accuracy of the prick test, which is not 100%, some cases of allergy may have been missed. Third, we exclude more than 45 cases due to missing data in their medical files, which requires another study with greater sample size to evaluate different food allergens in infants with higher statistically power. Finally, food allergy was assessed solely by skin prick testing (SPT). Although SPT is a useful and accepted diagnostic tool, it is more indicative of sensitization than of clinically confirmed allergy. The absence of confirmatory tests, such as serum specific IgE levels or oral food challenge may have led to an overestimation of the true prevalence of food allergy. The present study is that the assessment of food allergy was based solely on SPT, which primarily detects IgE‐mediated sensitization. However, we know that AD consists of two main pathophysiological subtypes: extrinsic AD (IgE‐mediated) and intrinsic AD (non‐IgE‐mediated). Patients with intrinsic AD may exhibit typical clinical features of the disease despite normal total IgE levels and negative SPT results, and therefore cannot be adequately identified using this diagnostic approach [42]. Consequently, the findings of this study largely reflect food allergen sensitization in infants with extrinsic AD and may underestimate the contribution of food‐related triggers to non‐IgE‐mediated disease. Future studies using additional diagnostic tools, such as serum biomarkers, elimination diets, and oral food challenges, are needed to better characterize intrinsic AD and its association with food sensitivity.

Future studies should be prospective, multicenter, and with larger sample sizes to improve generalizability and statistical power. Also, the use of standardized severity scoring systems for AD and confirmatory diagnostic methods such as serum IgE and oral food challenge may allow for more accurate identification of clinically relevant food allergies. Furthermore, we suggest that longitudinal studies are needed to clarify the temporal relationship between early‐onset AD, food sensitization, and disease severity, and to better understand the progression of allergic disease during infancy.

## 5 Conclusion

In the present study, infants with AD were most allergic to egg, cow's milk, and nuts, and also, the allergy to egg was significantly higher in girls, and cow's milk was significantly higher in infants with an age at onset of clinical manifestations lower than 6 months. Moreover, age at onset of clinical manifestations in severe AD cases was significantly lowest as compared to other severity levels. Identifying these allergens, avoiding them, and choosing conscious diets, along with drug treatment, play a very important role in the control and treatment of infants with AD. Allergic diseases and food sensitivities are increasing worldwide. Among AD patients, sensitivity to food allergens such as eggs, cow's milk, and wheat is common. Considering the onset of food sensitivity in many patients before 6 months of age, it is recommended that allergenic substances such as egg yolk and wheat should not be used for feeding infants before 6 months of age.

## Author Contributions

**Seyyed Ramin Madani:** methodology, conceptualization, supervision. **Mahdi Salehi:** investigation, software, data curation, writing – review and editing. **Marzieh Heidarzadeh Arani:** conceptualization, methodology, validation. **Zahra Shaterian:** project administration. **Mohammad Mahdi Heidari:** writing – review and editing, project administration, writing – original draft, visualization. **Mohammad Javad Azadchehr:** formal analysis.

## Conflicts of Interest

The authors declare no conflicts of interest.

## Supporting information


Supporting File 1

Supporting File 2

## Data Availability

The data that support the findings of this study are available from the corresponding author upon reasonable request. All relevant data generated or analyzed during this study are included in this published article. Additional data are available from the corresponding author on reasonable request.
