# Supplementary material for: Assessment of Common Food Allergens in Infants With Atopic Dermatitis in Kashan From 2005 to 2021
Source: Immun Inflamm Dis. 2026 Oct 6;14(10):e70544. doi: 10.1002/iid3.70544 (PMC13640520; doi:10.1002/iid3.70544)
Supplement: Supplementary file 2 [file IID3-14-e70544-s002.docx]

# STROBE Checklist for the Article

| Item | STROBE Criteria | Response (Based on the Article) |
| --- | --- | --- |
| Title and Abstract | Indicate the study’s design with a commonly used term in the title or the abstract. | The study is described as a retrospective case-series in the abstract. |
| Introduction | Explain the scientific background and rationale for the investigation. | The introduction provides a thorough background on atopic dermatitis and food allergies. |
| Methods - Study Design | Present key elements of study design early in the paper. | The study is a retrospective case-series covering 98 infants from 2005 to 2021. |
| Methods - Setting | Describe the setting, locations, and relevant dates. | The study was conducted at Kashan Shahid Beheshti Hospital in Iran from 2005 to 2021. |
| Methods - Participants | Give the eligibility criteria and the sources and methods of selection. | Infants aged 6-24 months with AD who underwent skin prick tests were included; exclusion criteria were also specified. |
| Methods - Variables | Clearly define all outcomes, exposures, predictors, potential confounders, and effect modifiers. | The study focuses on demographic variables, food allergens, and AD severity. |
| Methods - Data Sources | For each variable of interest, give sources of data and details of measurement. | Data were extracted from medical files, and skin prick tests were performed according to standard procedures. |
| Methods - Statistical Analysis | Describe all statistical methods, including control for confounding. | SPSS v24 was used for data analysis with Chi-square, t-tests, and ANOVA applied. |
| Results - Participants | Report numbers of individuals at each stage of study. | A flowchart (Figure 1) shows how 98 cases were selected from an initial 144 records. |
| Results - Descriptive Data | Give characteristics of study participants. | Tables provide details on gender distribution, nutrition type, and AD severity. |
| Results - Outcome Data | Report numbers of outcome events or summary measures. | Egg and cow’s milk were the most common allergens; statistical significance was reported. |
| Discussion - Key Results | Summarize key findings. | Egg and cow’s milk allergies were prevalent; early onset was linked to severity. |
| Discussion - Interpretation | Discuss results in relation to existing evidence. | Findings are compared with international and Iranian studies on food allergies in AD patients. |
| Funding | State funding sources and any role in the study. | Acknowledgment section states no conflict of interest and funding sources. |
